# Translational milestones in urologic pathology: integrating molecular diagnostics across cancer types

**DOI:** 10.1002/2056-4538.70046

**Published:** 2025-09-02

**Authors:** Andres Matoso, Andres M Acosta

**Affiliations:** ^1^ Department of Pathology The Johns Hopkins Medical Institutions Baltimore MD USA; ^2^ Department of Oncology The Johns Hopkins Medical Institutions Baltimore MD USA; ^3^ Department of Urology The Johns Hopkins Medical Institutions Baltimore MD USA; ^4^ Department of Pathology Indiana University Indianapolis IN USA

**Keywords:** renal cell carcinoma, Wilms tumor, prostate cancer, bladder cancer

## Abstract

In its first decade, *The Journal of Pathology: Clinical Research* has become a leading source of translational studies advancing molecular diagnostics in cancer, particularly in urologic pathology. This commentary highlights recent contributions that collectively place precision oncology at the forefront of pathology research. One review examines cancer stem cells in renal cell carcinoma, emphasizing the complexity of cellular plasticity and the tumor microenvironment in driving resistance and recurrence. In prostate cancer, epithelial‐to‐mesenchymal transition (EMT) regulators, including Twist, Slug, and Snail, are identified as synergistic markers of poor prognosis, linked to hypoxia and invasiveness. Another review details the integration of homologous recombination repair gene testing into clinical workflows, supporting targeted treatment strategies with poly (ADP‐ribose) polymerase inhibitors. In pediatric oncology, *TP53* alterations in Wilms tumor are shown to occur beyond anaplastic cases, expanding their prognostic significance. Advances in molecular subtyping are also demonstrated in bladder cancer, where transcriptomic profiling could enable tailored neoadjuvant therapy. In clear cell renal cell carcinoma, re‐evaluation of a prognostic model revealed that while necrosis or sarcomatoid differentiation correlated with poor outcomes, only DNA methylation markers improved prognostic accuracy, underscoring their utility for biopsy‐based risk stratification. Finally, digital spatial profiling of sarcomatoid urothelial carcinoma reveals an immunosuppressive microenvironment with CD163‐positive cells, implicating them in EMT and aggressive phenotype. Together, these studies highlight the transformative role of integrated molecular diagnostics in guiding individualized therapies and improving outcomes in urologic cancers.

In its first decade, *The Journal of Pathology: Clinical Research* has built a strong reputation for high‐quality translational research. Its most impactful studies and reviews highlight advances in molecular diagnostics, biomarker discovery, and cancer pathobiology, especially the roles of the tumor microenvironment (TME) and cellular plasticity in understanding cancer and developing new therapies.

The review by Corrò and Moch provides a comprehensive and insightful exploration into the complex landscape of cancer stem cells (CSCs) in renal cell carcinoma (RCC), emphasizing the critical need for precise biomarkers to improve diagnostics and treatment [1]. RCC, particularly clear cell RCC, is notoriously heterogeneous and resistant to conventional therapies, making CSC‐targeted research promising. The article outlines two fundamental models of tumor development, the clonal evolution and CSC (hierarchical) models, before advocating a unifying model that incorporates cellular plasticity and TME influences. This framework is essential to understand the dynamic and adaptive nature of CSCs, particularly in response to therapy and environmental pressures. The authors review a broad array of potential CSC markers in RCC, including CD105, CD133, CD44, CD24, CXCR4, and ALDH1. CD105 emerges as a particularly promising candidate, with evidence supporting its association with self‐renewal, angiogenesis, and tumorigenesis. However, the authors also highlight significant limitations in marker reliability. For instance, CD133's role remains contentious due to conflicting evidence about its tumorigenic potential, possibly because it marks normal renal progenitor cells rather than true CSCs. Importantly, the review underscores the role of the TME in sustaining CSC traits through mechanisms such as hypoxia‐induced signaling and immune evasion. Exosomes released by CSCs, which can modulate the immune response and promote metastasis, are discussed as both a challenge and a potential biomarker source. Isolation techniques are critically analyzed, including antigen‐based methods (fluorescence‐activated cell sorting, magnetic‐activated cell sorting), side population analysis, and 3D culture assays like sphere formation and organoid development. The authors note that while these approaches have advanced our understanding, the technical limitations of the assays and the intrinsic variability of CSCs impede the establishment of universal biomarkers. A key strength of the article lies in its call for integrated strategies that consider both molecular profiling and functional assays, alongside improved *in vivo* models incorporating humanized environments. The conclusion persuasively argues that therapeutic success hinges not only on identifying CSC markers but also on targeting CSC‐specific pathways and their supportive niches. In summary, Corrò and Moch present a balanced yet critical perspective on the promise and pitfalls of CSC biomarker discovery in RCC, advocating for multidimensional approaches to overcome the inherent complexities of cancer biology.

The study by Børretzen and colleagues delivers compelling evidence on the prognostic role of epithelial‐to‐mesenchymal transition (EMT) regulators, Twist, Slug (Snai2), and Snail (Snai1), in prostate cancer [2]. EMT is a critical process enabling epithelial tumor cells to acquire mesenchymal traits, facilitating invasion and metastasis [3]. The research underscores the relevance of these transcription factors in linking EMT, hypoxia, and angiogenesis to disease progression and survival outcomes. Using immunohistochemical analysis of 338 radical prostatectomy specimens, alongside cases of hyperplasia, metastases, and castration‐resistant prostate cancer, the study provides a comprehensive overview of the expression patterns of proteins implicated in EMT. Notably, strong expression of Twist and Slug was positively correlated with HIF‐1α, a key hypoxia marker, highlighting the interplay between hypoxic signaling and EMT regulation. A key insight is that high levels of Twist, Slug, and Snail (particularly at the tumor‐stromal interface) correlated with aggressive pathological features, such as high Gleason grade, extra‐prostatic extension, and seminal vesicle invasion, as well as shorter time to recurrence and cancer‐specific death. Importantly, the co‐expression of these EMT regulators had a cumulative negative effect on prognosis, indicating a synergistic influence on tumor aggressiveness. The study also distinguished the predictive value of Slug in E‐cadherin‐low tumors, where it emerged as an independent prognostic marker for cancer‐specific death. This highlights the heterogeneity of EMT as a process and suggests the potential utility of combining EMT markers with epithelial markers like E‐cadherin to refine prognostication.

Homologous recombination repair (HRR) deficiency has been linked to poor response to standard therapies in prostate cancer [4, 5, 6]. Gonzalez *et al* provide a timely review on the integration of HRR mutation testing into the clinical management of metastatic castration‐resistant prostate cancer (mCRPC) [7]. With the recent approval of poly (ADP‐ribose) polymerase (PARP) inhibitors such as olaparib and rucaparib for patients with HRR gene alterations, the article addresses the urgent need to optimize molecular diagnostics to guide targeted treatment strategies. This review paper comprehensively covers the biological rationale for HRR testing, explaining how defects in this DNA repair pathway, especially in genes like *BRCA1*, *BRCA2*, and *ATM*, render tumor cells susceptible to PARP inhibitors. Importantly, the authors underscore that approximately 25% of patients with mCRPC harbor HRR gene alterations, reinforcing the clinical utility of molecular stratification. A major contribution of this review is its in‐depth analysis of pre‐analytical and analytical challenges in implementing HRR testing. The authors identify critical barriers such as inadequate tumor content, degraded DNA in archival samples, and variation in tissue processing, which can lead to high test failure rates. To address this, they propose a series of practical recommendations, ranging from sample collection and fixation protocols to quality control in DNA extraction and sequencing methodologies. Particularly valuable is the emphasis on a multidisciplinary, patient‐centered approach involving urologists, oncologists, pathologists, radiologists, and laboratory staff. The review provides step‐by‐step guidance on how to identify, store, and process biopsy samples optimally for next‐generation sequencing. The authors also discuss the evolving role of circulating cell‐free DNA as a noninvasive alternative for HRR profiling when tissue samples are inadequate. Moreover, they draw attention to the ethical and clinical implications of germline findings uncovered through somatic testing emphasizing appropriate patient counseling and referral to genetics specialists, highlighting the broader impact of HRR mutations on family members and hereditary cancer risk.

Anaplasia has consistently been linked to poorer prognosis in patients with Wilms tumor (WT) compared to those with favorable histology, and current treatment protocols still rely on histologic classification for risk stratification [8, 9, 10]. It is thought to arise as a clonal event within tumors initially classified as favorable histology, based largely on findings that *TP53* mutations were present only in anaplastic regions of the same tumor [11, 12]. The study by Wegert *et al* explores the significance of *TP53* alterations in WT, with a particular focus on their relationship to anaplasia and clinical outcomes. The authors challenge the prevailing notion that *TP53* mutations are exclusive to anaplastic WTs by demonstrating that such alterations also occur in a significant subset of nonanaplastic but fatal cases. This finding has significant implications for diagnostic and prognostic strategies in pediatric oncology. By analyzing 84 fatal WT cases alongside a control group of surviving patients, the study presents compelling evidence that *TP53* mutations are nearly ubiquitous in diffuse anaplastic WT (DA‐WT), being found in 97% of fatal DA‐WT cases. However, the mutation frequency was similar in nonfatal DA‐WT, suggesting that the presence of *TP53* mutations alone does not predict outcome in the anaplastic group. Instead, tumor stage, particularly stage > I, emerged as a more reliable prognostic indicator. Importantly, 26% of nonanaplastic fatal tumors also harbored *TP53* mutations, often in samples that exhibited ‘nuclear unrest,’ hinting at anaplasia‐like features. This suggests a continuum rather than a dichotomy in WT histopathology and raises the possibility that screening for *TP53* alterations could help identify high‐risk cases otherwise classified as intermediate or low risk. A major strength of the study lies in its multi‐modal approach: integrating DNA sequencing, copy number variation analysis, and p53 protein immunohistochemistry. This multi‐pronged assessment confirmed p53 dysfunction in many cases and revealed striking intratumor heterogeneity. Through detailed spatial sampling, the authors show that *TP53* mutations tend to localize in anaplastic regions, reinforcing the notion that these are late events in tumor evolution. The findings highlight a significant challenge: the late emergence and heterogeneity of *TP53* alterations limit their clinical utility as a prognostic biomarker, particularly in routine diagnostics where biopsy sampling may miss critical regions. Nevertheless, the strong association between *TP53* status and anaplastic morphology suggests a potential role for *TP53* as a biomarker, especially when used in conjunction with histological assessment.

Advances in genomic technology have allowed for bladder tumors to be stratified into molecular subtypes (i.e., luminal, basal, infiltrated, squamous, neuronal, genomically unstable) [13, 14, 15]. The study by Griffin *et al* offers a critical foundational step for the GUSTO clinical trial, which aims to tailor neoadjuvant therapy in muscle‐invasive bladder cancer based on molecular subtypes [16]. By verifying the reproducibility and robustness of gene expression profiling using the Decipher Bladder platform and the cancer genome atlas (TCGA) subtyping model, the authors reinforce the feasibility of implementing precision oncology in a real‐world, multi‐center clinical setting. The verification process, involving 18 formalin‐fixed paraffin‐embedded (FFPE) bladder tumor samples, demonstrates high intra‐ and inter‐laboratory concordance in subtype assignment across technical replicates. This finding is particularly noteworthy given the use of RNA extracted from archival FFPE blocks, a known challenge due to RNA degradation. The successful subtyping despite moderate degradation [median DV200 (percentage of RNA fragments >200 nucleotides in size) of 45.5%] underscores the assay's resilience and the practicality of applying transcriptomic profiling in routine histopathology workflows. Importantly, the study not only establishes technical reproducibility but also reveals substantial morphological heterogeneity within subtypes, emphasizing that histological appearances alone are insufficient to predict molecular classification. This strengthens the rationale for molecular subtyping as a tool to stratify treatment, especially since existing retrospective studies suggest subtype‐dependent responses to neoadjuvant chemotherapy (NAC). For example, basal/squamous subtypes tend to benefit more from NAC, while luminal/papillary tumors show less responsiveness. The GUSTO trial aims to use this knowledge to direct therapy, potentially avoiding ineffective treatment and reducing toxicity for some patients. One of the key strengths of this study is its attention to real‐world variables such as RNA stability, block storage conditions, and laboratory variation. Despite these variables, all samples passed quality control and yielded valid subtyping results. This level of rigor provides confidence that molecular subtyping can be implemented effectively in decentralized clinical trial settings. However, a limitation lies in the small sample size of the verification cohort. While the authors acknowledge this, they also demonstrate that all relevant subtypes were represented, and external validation was conducted, mitigating concerns about generalizability [16].

Unlike tumor cells, stromal cells in the TME are genetically stable, making them attractive therapeutic targets [17]. The study by Johnson *et al* highlights the use of digital spatial profiling to uncover molecular distinctions between sarcomatoid and conventional components of urothelial carcinoma (UC), offering insights into the aggressive behavior of sarcomatoid urothelial carcinoma (SUC). By spatially analyzing gene expression across different tumor regions within the same specimens, the authors identified significant differences in the TME. SUC regions demonstrated higher stromal infiltration, particularly of fibroblasts and CD163‐positive macrophages, compared to conventional UC areas. Pathway analysis revealed enrichment of extracellular matrix‐related genes and elevated expression of transforming growth factor‐beta (TGFβ), a cytokine known to promote EMT and tumor progression. Immunohistochemistry confirmed that CD163‐positive macrophages associated with immunosuppressive, pro‐tumor activity were more abundant than CD68‐positive macrophages in SUC regions. These findings support a model in which immune‐suppressive macrophages and fibroblasts contribute to EMT and drive the aggressive phenotype of SUC [18].

Finally, Odeh *et al* re‐evaluated a prognostic model for clear cell renal cell carcinoma (ccRCC), combining five DNA methylation markers (*GREM1*, *GATA5*, *LAD1*, *NEFH*, and *NEURL*) with clinicopathological features [19]. Using ISUP/WHO 2022 criteria and the TNM 8th edition, they found previously that pathological features (necrosis, lymphovascular invasion, sarcomatoid and rhabdoid differentiation) were linked to poorer outcomes but did not improve multivariate model performance [20]. The updated model performed similarly to the original, whereas adding DNA methylation markers consistently enhanced prognostic accuracy, highlighting their potential for improving risk stratification, particularly in biopsy‐based assessment [19].

As *The Journal of Pathology: Clinical Research* enters its second decade, the studies highlighted here underscore a transformative era in urologic pathology, where molecular diagnostics are not only enhancing our understanding of disease mechanisms but increasingly guiding clinical decision‐making (Figure 1). From RCC to prostate and bladder cancer, the integration of biomarkers in routine practice is driving a paradigm shift toward precision medicine. Looking ahead, the future of urologic pathology lies in harnessing these molecular insights to deliver more tailored, effective therapies. The convergence of transcriptomic profiling, genomic sequencing, and advanced *in vivo* models will enable a deeper understanding of tumor heterogeneity and treatment resistance. At the same time, functional assays and real‐time liquid biopsy tools like circulating tumor DNA are poised to complement traditional tissue‐based diagnostics, offering minimally invasive ways to monitor disease progression and therapeutic response.

**Figure 1:**
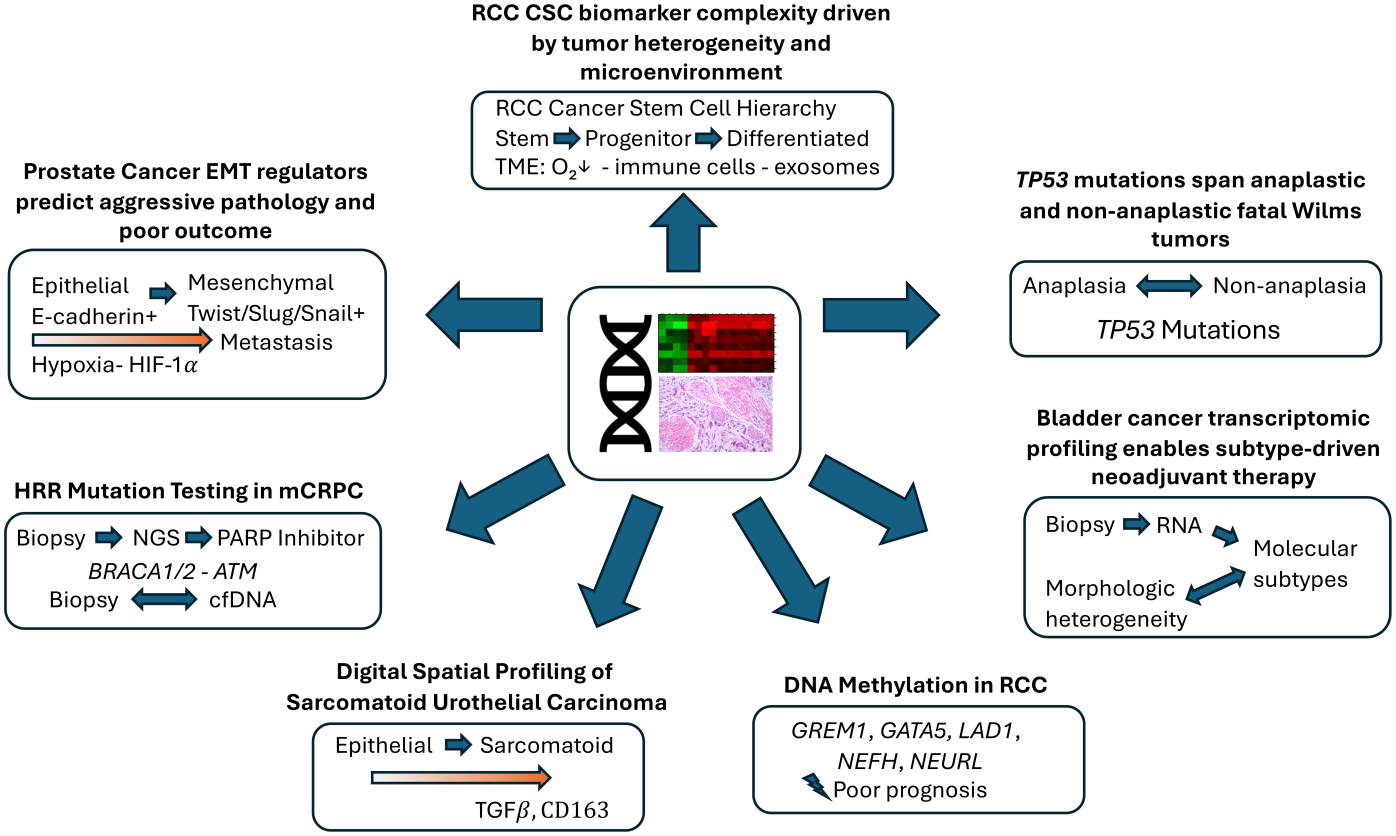
This multi‐panel schematic illustrates key advancements in precision oncology across urologic malignancies, highlighting the integration of molecular diagnostics to better understand tumor biology and identify potential therapeutic targets.

## Author contributions statement

AM designed and drafted the manuscript. AMA reviewed and edited the manuscript.
